# "Part of the Solution": Food Corporation Strategies for Regulatory Capture and Legitimacy

**DOI:** 10.34172/ijhpm.2021.111

**Published:** 2021-09-11

**Authors:** Jennifer Lacy-Nichols, Owain Williams

**Affiliations:** ^1^Centre for Health Policy, Melbourne School of Population and Global Health, University of Melbourne, Melbourne, Australia.; ^2^School of Politics and International Studies, University of Leeds, Leeds, UK.

**Keywords:** Food Industry, Corporate Power, Corporate Social Responsibility, Hegemony

## Abstract

**Background:** For decades, the food industry has sought to deflect criticisms of its products and block public health legislation through a range of offensive and defensive strategies. More recently, food corporations have moved on to present themselves as "part of the solution" to the health problems their products cause. This strategic approach is characterised by appeasement, co-option and partnership, and involves incremental concessions and attempts to partner with health actors. This paper details how corporate practices have evolved and changed over the past two decades and gives some definition to what this new political economy signifies for the wider behaviours of corporations producing and selling harmful commodities.

**Methods:** This paper draws on public health and political science literature to classify the food industry’s "part of the solution" strategy into three broad components: regulatory responses and capture; relationship building; and market strategies. We detail the key characteristics and consequences of each component.

**Results:** The three components of the food industry’s "part of the solution" strategy all involve elements of appeasement and co-option. They also improve the political environment and resources of the food industry. Regulatory responses offer incremental concessions that seek to maintain corporate influence over governance processes and minimise the threat of regulations; relationship building fosters access to health and government stakeholders, and opportunities to acquire and maintain channels of direct influence; and market strategies to make products and portfolios healthier bolster the market share and revenue of food corporations while improving their public image.

**Conclusion:** Rather being a signal of lost position and power, the food industry’s repositioning as "part of the solution" has created a highly profitable political economy of ‘healthy’ food production, alongside continued production of unhealthy commodities, a strategy in which it is also less burdensome and conflictual for corporations to exercise political power and influence.

## Background

The response and positioning of the food industry to obesity and other diet-related diseases has substantially changed since the early 2000s. Before this point of transition, the corporate strategic response to public health criticisms of their products and government attempts to regulate their industries were vigorously oppositional. Burgeoning criticism of their products and business models, increasingly oppositional government and civil society, and attempts to regulate products and markets, were all met with countervailing force.^1-4^ Indeed, the food industry had historically deployed a range of offensive and defensive political strategies to protect its business interests. Public health researchers have analysed these strategies, and collectively this body of literature illuminates the packaged food and beverage industry’s more overt and confrontational efforts to shape the political, regulatory and non-market environment in its favour.^5-9^ These offensive and defensive strategies have marked the histories of corporate strategies toward potentially hostile regulation of harmful products in general and have three broad dimensions.

The first involves attacking, distorting or undermining the considerable bodies of scientific evidence linking consumption of particular products to obesity or other diet-related diseases, for example by funding research that disputes the relationship between sugary drinks and obesity.^10^ The literature on the corporate use of science denial has grown, and now encompasses many decades of subterfuge by both the food and tobacco industries. Funding ‘astroturf’ groups and think tanks (such as the Heritage Foundation) to oppose policy and science, and the deployment of ‘fake scientists’ and suborned expertise have also been commonly used and documented, as for example by the Big Sugar industries.^11^

A second strategy focused on manipulating the mechanisms and agents of policy-making, using political donations, special interest pleading for the benefits of associated agriculture for farmers groups and so on, or by using political influence to block or undermine public health policies perceived as threatening to business interests, as has been the case with the stymieing the development of robust nutrition policies in national and multilateral contexts.^5^ Campaign donations to influence voting, exercising influence through the revolving door, were routine and still very much continue even after the ostensible shift in corporate strategy toward public health that we detail.^12^ The criticism of public health policies and use of litigation to oppose unwanted policies have also been used been offensively and defensively to measures that would affect market positions or sale of harmful products.^13,14^

A third strategy sought to fragment and discredit industry opposition within the public health nutrition community.^15,16^ This has included personal threats to researchers and public health professionals^15,17^ as well as monitoring and surveillance of them by corporations.^18,19^ Companies have also used their public relations divisions and corporate social responsibility (CSR) initiatives to position themselves as public health leaders. For example, internal documents from Coca-Cola reveal that an explicit aim of its “Movement is Happiness” campaign was to “marginalize detractors,” namely those in the public health community and government that were opposed to its core market strategies and outlook.^20^ Public health advocacy more generally has therefore suffered, and been described as perhaps consequentially fragmented and often uncoordinated.^21,22^

Of course, all these strategies are not mutually exclusive and have been deployed simultaneously across multiple sectors, products, institutions and scientific and regulatory domains. We note that is a multi-level and multi-nodal governance strategic ‘game’ that corporations have become adept at playing across different governance settings and spheres of public life, using techniques such as forum shifting to pursue gains.^23^ However, we argue that something has changed, and it is clear that the business and non-market strategies of food corporations have reconfigured themselves over the last two decades in terms of the use of different levers and strategies that are now employed to achieve the regulatory and non-market political and regulatory conditions. These political and regulatory conditions – the non-market environment - form an essential counterpart to their market strategies and profits. Indeed, since 2000 even the defensive and oppositional strategies described above have become more nuanced in parallel with the approaches described in this paper. Food corporations now additionally pursue what can be framed as a “part of the solution” strategy, characterised by appeasement and co-option (in contrast to the overt opposition which characterised their early strategies). In this paper we seek to outline how the strategic game had shifted and why. What is immediately clear is that food corporations have recognised that scientific evidence of the health harms being caused by their products, such as ultra-processed, sugar sweetened, high-salt content and trans-fat laden foods, was simply too overwhelming to counter simply by continuing to rely upon strategies of opposition, doubt and denial. Growing public awareness of the health harms associated with consumption of ultra-processed foods presented a business risk for the food industry (for example as evidenced in reduced sales of sugary drinks in the Global North) as well as a potential loss of credibility, as public health campaigns and media attention exposed the food industry’s political strategies.^24-26^ Furthermore, by the early 2000s, governments and multilateral bodies were finally acting against their products and business strategies by means of legislation, or by real or threatened hostile regulatory environments. In response, food corporations have moved on to present industry as being a necessary “part of the solution” to the health problems their products cause.

The spectrum of industry strategies that fall under what can be loosely termed the “part of the solution” umbrella is broad, and available analyses differs in terms of what are included or excluded from typologies of this strategic approach.^5,16,27,28^ Among the strategies of co-option and appeasement in the food domain we would point to the importance of product reformulation, nutrition labelling, restricting marketing to children, nutrition education, the removal of unhealthy products from schools and promotion of physical activity. These are both important marketing strategies and non-market strategies that give legitimacy to the firms involved, creating, again, a perception of good health citizenship. Added to this, corporations are more engaged in public-private partnerships and the levers that ensure that regulation stops at self-regulation. As was the case with the offensive and defensive strategic outlook, these areas of strategy are pursued as a simultaneous, multi-level, multi-nodal manner, requiring engagement with other actors over a range of institutions, products and sectors.

It is significant that the “part of the solution” strategy first emerged in the early 2000s, heralded by public pledges from leading food companies about how they would help address obesity. In early 2003, Kraft Foods publicly acknowledged the role of the food industry in the obesity ‘issue,’ and later that year Coca-Cola released its first “Model Guidelines for School Beverage Partnerships.”^29,30^ Beneath these ‘Road to Damascus changes’ several market and political dynamics actually compelled the food industry to shift strategy. Of these dynamics, the changing logics of corporate risk and threatened market positions loom large. For example, concerns from business investors about the risks obesity presented for some food companies have been identified^31,32^; as well as a growing body of research about the contribution of the food environment to diet-related diseases.^33-36^ Furthermore, campaigns from civil society and public health organisations targeting the food industry were gaining traction.^5^ Notably, alongside these shifts in the calculations of risk, causation and accountability, we see the emergence of government policies regulating the food industry, and in particular soft drinks.^37^

But industry is agile and thinks ahead (hence strategy), while learning the lessons of past mistakes by other sectors. The food industry learned from the tobacco industry. The timing of the strategic shift with food corporations follows a significant transition in public, political and regulatory orientations regarding another harmful product sector, where corporations had by then over-played their hand in terms of the strategies of opposition and hostility described above. By the late 1990s, tobacco companies became concerned about their loss of access to political elites and the dangers of hostile regulatory environments and loss of regulatory chill, as epitomised in the development of the Framework Convention on Tobacco Control. Big Tobacco then began a CSR campaign to win back access to policy-makers and improve their public image,^38^ a strategy that continues today. Reflecting on the tobacco industry’s previous strategy of denial and hostility, a former Vice President of Kraft (which was part of tobacco company Philip Morris for two decades) argued that the tobacco industry would have been better off reforming their marketing and sacrificing some profits in order to “disarm their critics.”^39^ In light of the tobacco industry’s experience, we can observe that a key aim of the food industry’s “part of the solution” strategy has been to maintain political legitimacy by means of appeasement and engagement. It is also notable that many of the front foundations and scientists employed by tobacco in their denial and doubt campaigns migrated to food sectors, bringing further knowledge of necessary adjustments to the new political environment with them.^40^

This paper seeks to show how this “part of the solution” strategy has emerged and diffused through the food industry in the context of growing market and regulatory threats to the industry. We note from the outset that more offensive and oppositional strategies are still part of food industry non-market strategies, especially when core market positions are under threat, or when regulatory, legal or food governance arrangements are perceived as weak or easy to dominate, as is often the case in some low-and middle-income countries and the United States.^41,42^ While corporations will therefore also continue to sell harmful products where and if they can, they have also switched market strategies to diversify products while developing non-market strategies to accommodate and co-opt healthy eating agendas and public health advances. New, more agile market strategies and forms of relationship building with regulators and stakeholders have been advanced.

 We seek to detail how the food industry’s “part of the solution” strategy is characterised by agility and responsiveness, as seen through three key pillars of this strategy: regulatory responses and capture; relationship building; and new market strategies. These three strategic pillars also help reveal and delineate the strategic value of appeasement and co-option (as opposed to overt opposition), and we give some definition to what this new political economy signifies for the wider behaviours of corporations producing and selling harmful commodities. We argue that rather being a signal of lost position and power, the development of a new strategic platform has actually created new business opportunities emerging from new products and markets. Of course, it has also acted to salvage an otherwise potentially catastrophic regulatory environment. Instead, the change of strategy has created an environment less hostile to business interests than appeared to be emerging at the turn of the millennium, arguably fostering a transformed political and regulatory environment in which it has become less burdensome and conflictual for corporations to exercise political power and influence.

## Methods

This paper offers a theoretical and critical public health discussion of the food industry’s strategy to present itself as ‘part of the solution’ to obesity. It draws on political science and food policy scholarship, in particular the concepts of hegemony and corporate power, to interpret the shift in food corporation strategies from overt opposition and conflict to appeasement and compromise. Key elements of hegemony relevant to our analysis are grounded in Gramscian notions that “coalitions and compromises that provide a measure of political and material accommodation” in order to preserve the existing status quo, and that power is amplified and legitimised when it is garnered by what are perceived as being socially consensual means.^43,44^ Furthermore, food policy scholarship has similarly argued that powerful corporations “appropriate social movement demands to serve renewed accumulation,” thus bringing in Gramscian understandings of co-option as a tool of hegemonic power.^45^ It is through these lenses of *accommodation, consensus and co-option in the pursuit of profits and power* that we interpret the food industry’s strategy to position itself as part of the solution. We will show that while many corporate initiatives in these directions, at face value, show the food industry’s willingness to make incremental changes to align with public health demands, they also serve to reinforce the industry’s economic and political power, and do so with a semblance of perceived legitimacy and notwithstanding the market strategies and health harms they continue to perpetuate.

 We have selected three facets of the food industry’s ‘part of the solution’ strategy to demonstrate how accommodation and co-option manifest across different market and political activities of the global food industry: regulatory responses, relationship building and market strategies. The first strategy concerning regulatory responses is an example of how food companies and trade associations have sought to capture, co-opt and diminish regulatory processes through the use and promotion of industry self-regulation. The second strategic response, relationship building, demonstrates how the food industry has strategically cultivated partnerships with credible stakeholders in an effort to bolster their legitimacy through co-option and consensus building. The third, new market strategies, reveals how food companies have changed their product portfolios to more closely align with public health recommendations, whilst disarming criticism and making profit.

 For each case, we outline the characteristics of each component and provide illustrative examples of how some of the largest global food companies and trade associations have implemented these strategies over the past two decades. These examples are drawn from the published literature and from primary research conducted by the authors. The examples are not intended to be comprehensive, but rather illustrate the breadth and diversity of activities that the largest food industry actors have engaged in. Where possible, examples are provided for a range of countries in the global North and South, as well as national and global initiatives.

 We conclude with a discussion of the broad contours of the food corporate strategic culture of accommodation that as emerged in the last two decades and how it differs from and supports its strategy of opposition. Our discussion explores what these dynamics signal for the political economy of food markets and their governance.

## Results

###  Regulatory Responses

Corporate self-regulation is a key component of the food industry’s strategy to position itself as “part of the solution” to obesity and other diet-related health issues. Self-regulation refers to the voluntary pledges, commitments, standards, codes of practice or other rule-setting initiatives that companies or industries have created and pledged to follow.^46-48^ The scope of self-regulation varies considerably between companies, countries and issues (see Table 1).

**Table 1:** Examples of Food Industry Self-regulation Initiatives

**Stakeholder**	**Year**	**Location**	**Focus**
Kraft Food	2003	United States	School marketing, portion size, product nutrition
Coca-Cola	2003	United States	Beverage availability in school vending machines
Kraft Food	2004	United States	Front of pack labelling, general health promotion
American Beverage Association	2005	United States	Beverage availability in school vending machines
International Council of Beverages Associations	2008	Global	Marketing to children
International Food and Beverage Alliance	2008	Global	Reformulation, marketing to children, labelling, education, public-private-partnerships
Australian Food and Grocery Council	2009	Australia	Marketing to children
Nestlé	2014	Global	Front-of-pack labelling
PepsiCo	2016	Global	Sugar reduction/reformulation
Australian Beverages Council	2018	Australia	Sugar reduction/reformulation
Unilever	nd	Thailand	Salt reduction/reformulation

Information sourced from:^21,29,48-51^

What is not immediately apparent from this table is that self-regulation initiatives are not static initiatives, but “living documents” and programs that change over time in response to changes in their political and market environments.^48^ Since the early 2000s, self-regulation has gradually transformed from relatively weak and uncoordinated corporate pledges to a coordinated sector-wide set of commitments and actions that respond to specific public health policy proposals. For example, Kraft’s 2003 pledge referred to improving existing products and capping the portion size of single serve packages, yet it did not provide details about what this would entail or any timeframes for achieving the pledge.^29^ In contrast, the company’s 2021 pledges provide specific nutrient reduction targets and timeframes in line with their ‘Global Nutrition Guidelines.’^52^ Situating the food industry’s gradual strengthening of its pledges and actions within an increasingly hostile regulatory landscape helps to understand some of the political benefits of adapting and improving self-regulation.

On the one hand, this transformation reflects the growing regulatory threats that the food industry faces, as governments develop a range of initiatives to reduce consumption of unhealthy foods and beverages.^37^ Rather than passively accept these policies, food companies have used and are using self-regulation to proactively pre-empt policy development and hostile regulatory environments. For example the International Food and Beverage Alliance launched its global pledge during the 2008 World Health Assembly where world leaders were planning to discuss the issue of marketing to children; and soft drink companies have launched sugar reduction pledges when governments begin to consider taxes or labelling polices.^53-55^

The ability to modify and re-launch an industry pledge/s helps the strategy of self-regulation to accommodate and pacify public health pressure for regulatory or legislative action. For example, the Australian Beverages Council sought to pre-empt a proposed legislated tax on sugary drinks by launching a reformulation pledge.^56^ In contrast, the failure to adapt and improve self-regulation may contribute to an otherwise more enabling environment for government regulation. While the food industry rolled out front-of-pack labelling schemes in the mid-2000s, these have remained relatively unchanged since their inception (for example soft drink industry calorie/energy labelling, or variations on the Daily Intake Guide for the packaged food industry).^57^ As governments become more proactive in developing policies to improve food environments in their countries, this food industry inertia may have also contributed to the success of stricter labelling schemes, such as with the multiple traffic light in the United Kingdom, Ecuador and Bolivia and the black stop sign in Chile.^58-60^ In contrast, the absence of mandatory reformulation targets in most countries suggests that the food industry’s more dynamic reformulation initiatives helped to diffuse pressure for regulation.^21,61^ In positioning self-regulation as a policy alternative, the food industry performs a balancing act. The industry pledge must demonstrate enough progress to meet some (but not all) of the demands from public health. This allows them to progressively incorporate additional demands from public health and launch modified pledge with the attendant benefits of public relations, policy pre-emption and chill (for example, Pepsi has launched several versions of its global commitment to reduce sugar in its products).^62,63^

Analysis of industry forestry certification standards also describes a similar “ratcheting up” of industry standards and suggests that concessions and accommodations are used to acquire and secure corporate rule-making power.^64^ The willingness of the food industry to continually adapt and change its self-regulation initiatives suggests that the key issue for the industry is not the debate over what goes into nutrition policies, but rather the debate over who governs nutrition policies. The development of self-regulation also represents the food industry’s attempt to create nutrition policy on its own terms and to demonstrate its capacity to participate, consensually and collaboratively, in policy development (a theme we elaborate on in the following section).

###  Relationship Building 

A second facet of the co-option and appeasement strategy is fostering or improving relationships with health and government stakeholders. The food industry pursues relationship building in a multitude of ways that go far beyond the traditional lobbying that occurs in the corridors of government. These relationships (and relationship building activities) differ in terms of their formality and institutionalisation, ranging from research contracts and formal advisory groups to more ad hoc and opportunistic activities, such as inviting politicians to attend board meetings. Table 2 documents a range of strategies that the food industry has used to initiate, maintain or improve relationships between the food industry and health and government stakeholders.

**Table 2:** Relationship Building Activities

**Industry Stakeholder**	**Activity**	**Public Health/Government Stakeholder**	**Year**
Australian Beverages Council	Government stakeholders invited speakers at Victorian Beverage Council Annual General Meeting	Australian Parliamentary Secretary to the Health Minister	2001
International Life Sciences Institute	Sponsor conferences, working groups and programs in China that promoted physical activity over nutrition	(Chinese) researchers; health officials; clinicians	2004-2015
Coca-Cola, PepsiCo and Cadbury-Schweppes	Develop the Alliance for a Healthier Generation to reduce the availability of sugary drinks in American schools	American Heart Association; Clinton Foundation	2006
Coca-Cola, PepsiCo	Sponsor 95 national health organisations in the United States	Centres for Disease Control, National Institute of Health, National Dental Association, American Diabetes Association (etc)	2011-2015
Australian Beverages Council	Government stakeholders invited to attend Australian Beverages Council annual board meeting	Independent Senator for South Australia; Parliamentary Secretary to the Prime Minister; CEO of Australian Chamber of Commerce and Industry; CEO of FSANZ, Deputy CEO of AFGC	2014
Nestlé	Collaborate with South African government to provide educational resources about “healthy eating”	Department of Basic Education (South Africa)	2018

Abbreviations: CEO, chief executive officer; FSANZ, Food Standards Australia New Zealand; AFGC, Australian Food and Grocery Council. Information sourced from:^30,65-69^

Some strategies focus more on building relationships with health professionals and researchers than on policy-makers or institutions, for example recruiting public health and nutrition professionals to serve as advisors or to conduct research for the industry.^3,5^ Relationships with health and nutrition professionals provide professional expertise and can serve as liaisons between industry and health communities, providing similar to the benefits of the revolving door with government agencies.^3,5,12^ The food industry has also participated in the evaluation of their self-regulation, providing data and information to researchers. For example, organisations that monitor corporate health promotion initiatives (such as the Access to Nutrition Index, INFORMAS (International Network for Food and Obesity/non-communicable diseases Research, Monitoring and Action Support) and the George Institute) often solicit information and data directly from the packaged food and beverage industry.^70-73^ These relationships foster the perception that the industry is cooperative and willing to facilitate monitoring of its own initiatives.

The food industry also seeks to gain access to policy-makers and the policy-making process, for example participating in government inquiries, writing submissions to policies, sitting on policy committees or (increasingly) working in partnership with governments through public-private-partnership platforms.^74,75^ The food industry has leveraged its other coalition building strategies to gain access to policy-makers and the policy-making process. For example, the public-private initiative Scaling Up Nutrition has enabled a large number of powerful companies to participate in the World Health Organization (WHO) policy deliberations and decision making.^76^ The development of self-regulation can be used as an opportunity to reach out to health organisations or government stakeholders for feedback on its development.^53,77^ Multiple political access points are especially important for industries that face exclusion from policy-making, such as was the case with the tobacco industry.^38^ As the soft drink industry faces growing scrutiny over its relationships with nutrition researchers and policy-makers, it may use corporate health promotion to broaden its connections with other stakeholders and expand its network of indirect political connections.^3,78-80^

In addition to growing its political allies and connections, appeasement and partnership strategies can be used together to garner praise from vocal industry critics as well. The legitimising function of co-opting erstwhile critics is clearly important. A striking example of this was the US Center for Science in the Public Interest’s praise for the confectionary company Mars when it announced its support for added sugar labelling.^81^ The Center for Science in the Public Interest is an outspoken industry critic that has sued the American soft drink industry for deceptive marketing.^82^ The potential for the appeasement strategies to foster approval from industry critics or generate allies for the industry therefore differentiates it from more oppositional political strategies. The possibility that antagonistic political strategies will damage the public reputation of a company is why we often see the food industry’s front groups be the agents that take a more oppositional stance, while the company presents a conciliatory position.^83^ Furthermore, the absence of oppositional strategies from CSR reporting and the industry’s general unwillingness to provide transparency around these strategies indicate that corporations recognise the public backlash these strategies can bring.^84^

While the proliferation of public-private partnerships is in part an indicator of the success of this strategy, this needs to be set in the context of a blurring between public and private forms of governance and the rise of public-private-partnerships as an increasingly common and acceptable form of governance.^85-88^ These forms of public-private governance be seen both at the national level (for example the Healthy Eating Partnership in Australia and the Public Health Responsibility Deal in the United Kingdom) as well as in international fora (for example the “Scaling Up Nutrition Movement,” the Global Alliance for Improved Nutrition).^86,89-91^ The process is two way. Public-private modes of governance in health socialise governments, health experts and advocates to business practices and interests, with the possibility that governance at least recognises or even promotes market interests; while at the same time it brings business in to governance rather than being purely its subject. Overall, this is part of the public-private thrust of health governance around the turn of the millennium, it becomes more business -like and more business friendly, with business at the table and part of the solution across a range of spheres of global health – from access to medicines through to nutrition and obesity prevention.^92^

###  Market Strategies

A third facet of the “part of the solution” strategy has been to improve products and product portfolios in terms of their health harming composition or production methods. This includes portfolio diversification as well as reformulation of products to improve their nutrient composition (eg, reducing sugar or adding vitamins and minerals).^93^ Like self-regulation, these initiatives differ enormously between products, companies, countries and industries. Table 3 documents some of the reformulation pledges made by members of the International Food and Beverage Alliance as well as “healthy” brand acquisitions to diversify the portfolios of processed food companies.

**Table 3:** Reformulation Pledges From IFBA Members

**Company**	**“Healthy” Brand Acquisition**	**Reformulation Commitment**
Kellogg’s	1999 acquire MorningStar Farms (veggie protein brand); launched Incogmeato burger in 2019 which will “bleed on the grill”	Reduce sodium in cereals on average by more than 30% Ensure that 90% of cereals have 10 g or less of sugar per 30 g serving
Danone	2001 acquire Stonyfield (organic brand)	100% of its products to meet the Danone Nutritional Targets by 2020
Coca-Cola	2011 acquire Honest Tea (organic brand)	Sugar reduced in more than 400 products
General Mills	2014 acquire Annie’s Inc. (organic brand)	Sugar reduction target of 5% or more and strategy to limit calories
Mondelez	2015 acquire Enjoy Life Foods (natural foods company)	Expand well-being brands in the portfolio, growing them at twice the rate of the base portfolio
PepsiCo	2018 acquire Health Warrior (plant-based nutrition company)	By 2025 –At least 3/4 of the global foods portfolio volume will not exceed 1.1g of saturated fat per 100 calorie
Unilever	2017 acquire Tazo Tea and Sir Kensington’s (organic brands)	By 2020 -Remove an additional 25% sugar in ready-to-drink teas
Mars	2019 acquire Foodspring (nutrition company)	Reduce sodium in the global portfolio by 20% by 2021

Abbreviation: IFBA, International Food and Beverages Association. Information sourced from:^63,94-98^

This focus on improving product quality is in part a reflection of the focus public health campaigns and policies, which are often designed with the explicit aim of encouraging changes to products (for example the UK’s levy on sugary drinks or design of front-of-pack labelling policies).^99-101^ However, while many public health professionals support reformulation in principle, they raise concerns about the food industry has (re)interpreted reformulation to suit its business interests. Key criticisms of corporate reformulation include that firms are actually developing new products rather than reformulation the original product; that reformulating only a selection of products and not all products in the company’s portfolio is common; that there is uneven progress across country jurisdictions; that only incremental changes to products are the norm; and finally to questions regarding the ingredients used to substitute for the “harmful” nutrient, for example in the use of artificial sweeteners or fats.^61,93,102^

These concerns focus mainly on the limited public health benefits of voluntary, industry-led reformulation initiatives. We can also consider some of the broader political and market consequences of both reformulation and diversification. Product reformulation and portfolio diversification more generally have expanded the food industry’s market share and revenue, as well as their penetration of developing markets.^103^ One study found that announcements of “healthy” new product launches improved the stock market performance of major food companies.^104^ The food industry’s response to health concerns about the products it sells has clear parallels with the food industry’s response to concerns about its environmental and labour practices. Like nutrition-oriented product reformulation, both organic and Fairtrade products have expanded corporate profits and market share.^45,105-108^ On the one hand, the win-win aspect of product reformulation highlights the success of the food industry in commercialising and profiting from public health solutions to obesity. However, on the other, the ease with which these new sites of profit can be transformed into political influence underscores the risks that market solutions to public health issues present more generally.^74^

We can also observe that, like the food industry’s development of organic and Fairtrade standards, the food industry uses reformulation to diffuse pressure for more radical changes to the production and consumption practices underpinning the current food system. Previous research has documented how Coca-Cola’s reformulation strategies in Australia have progressively aligned with public health expectations, shifting from “offering choices” to a more (albeit incomplete) systematic sugar reduction program across its portfolio.^61^ These incremental improvements in food quality may diffuse the market and political pressure driving companies to reformulate.^109^ Moreover, product reformulation circumvents the need for more radical initiatives that impact the availability and affordability of processed foods and beverages. Some of the radical changes feared by the food industry have been intimated in the scholarly literature, where Clapp and Scrinis^110^ note food companies “rarely entertain the notion of removing certain products from their lines altogether.” Fuchs et al^111^ note that food industry initiatives avert the need for absolute reductions in production and consumption. Product reformulation represents a minor concession that accommodates some of the nutrition community’s concerns while enabling the continued consumption of branded foods and beverages. Interestingly, while brand acquisitions garner attention in the market analyses, they are often camouflaged from consumers. While a small number of corporations dominate key sectors of the food system (for example meat processing, packaged food and drink, retailing), this is not always reflected on product labels—a phenomenon referred to as “stealth ownership.”^112^ While the diversity of foods on supermarket shelves may suggest a similarly diverse food system, in fact the food system is characterised by intense concentration of corporate ownership.^113,114^

## Discussion

Unlike corporate oppositional strategies, which are characterised by overt and aggressive contestation and threat-response reactions to political and policy pressures, the food industry’s part of the solution strategy is characterised by appeasement (eg, via incremental concessions) and co-option (eg, attempts to partner with health organisations and health actors). Food corporations have particularly sought to engage with the policy and fora to which they previously sought to oppose, recognising that being present at the table where agendas are set, and being generally a more engaged and open actor, are themselves strategically powerful moves.^74^ A seat at the table is after all both a voice and influence, a chance to set agendas and an opportunity to construct the ‘solution.’^115^ This new strategic outlook has also involved concessions being made to public health, such as in areas like product content, labelling or advertising. In short, corporate non-market strategy has moved toward the general promotion of industry as a ‘good citizen’ willing to adapt for health goals.

While appeasement of public health concerns is important enough, the process of engagement and relationship building has involved food corporations trying to reconstruct themselves as health actors, often co-opting the language and discourses of health and blurring their agency in new and rather profane ways. For example, we have seen a raft of health promoting strategies launched by food corporations, involving the stretched combinations of concepts and terms such CSR, social environmental and health, the triple bottom line, and CSR initiative names such “Help Hunger Disappear,” “Healthy Living” and “Good Food, Good Life Community Program.”^116^ Public health researchers have variously translated these as “health branding,”^117^ “public health CSR,”^77^ “CSR health-related activities,”^118^ corporate “health and wellness programs,” and “corporate health promotion.”^6^ Herrick^119^ is surely right in labelling these moves, collapsed terms and self-labelling as the “strategic appropriation of health by CSR;” but it is more than this. It reflects how corporations have sought to colonise processes, discourses and institutional forms of behaviour in health by emulation and assimilation. This is a strategy of co-option and hegemonic power consolidation which Gramscian scholars would readily identify.

While the offensive and defensive strategies of the past have perhaps tarnished the reputation of the food industry and fostered reciprocal opposition and antagonism from public health advocates, the food industry’s subsequent strategy to position itself as part of the solution to obesity and other diet-related diseases works to appease and pacify the public health community (although this is not always successful).^84,120^ But more widely, we must also remember that public health measures depend on political and sometimes public approval. Becoming legitimate and co-opting discourses, as well as diluting reputational damages of the past, is proving an exceptionally powerful tool of corporate reinvention and a profound recalibration of the perception of legitimacy of corporations with respect to public health actors.^121^ Consensual and socially legitimate power is far more powerful than that achieved through hostility and coercion, and far less burdensome in the medium to long term market strategies of corporations.

Indeed, scholars researching power differentiate between a coercive or compulsory exercise of power and a more persuasive exercise of power that is sometimes referred to as hidden, invisible or diffuse.^111,122-124^ The idea that power can have a coercive nature fits with descriptions of how power is exercised to resist or oppose unwanted political strategies—for example the soft drink industry’s threats of job losses to oppose taxes on their products.^125^ In contrast, we can think of the strategies of appeasement, co-option and partnership as a more persuasive and pervasive form of power. It might also prove more durable due to its co-option of epistemic and regulatory communities concerned with public health. Again, lessons can (and likely are) learned from other sectors engaged with harmful commodities or products. For example, in the late 1990s, for example, in response to the threat of regulation of emissions, the automobile industry began to invest in low-emission technologies and promoted a “win-win” rhetoric of environmental sustainability and corporate profits.^126^ This distinction between oppositional and appeasing manifestations of power helps to illuminate and explain some of the challenges and opportunities that the soft drink industry faces in using different political strategies. We can observe that oppositional manifestations of power are more likely to generate antagonism toward the industry, such as when the American and UK soft drink industries threated to withdraw investments or lay off employees if a sugary drink tax was passed (an exercise of oppositional structural power).^13,127^ In contrast, conciliatory manifestations of power are more likely to appease or “pacify” industry critics,^84,128^ such as when PepsiCo pledged to display calorie counts on front-of-pack labels (an exercise of conciliatory structural power), or in cases when the food industry participates in public-private partnerships (an exercise of conciliatory instrumental power).^110,129^ For example the soft drink industry’s partnership with the Clinton Foundation and American Heart Association in the United States helped to avoid pending litigation against the industry.^30^

The ability of corporate concessions to facilitate corporate governance of the food system reflects a core difference in how oppositional and conciliatory political strategies influence the political environment and generate a political economy of food in which corporate power and activities are normalised. Both the threat of job loss (an oppositional strategy) or the offer of concessions (an appeasement strategy) can delay the advent of unwanted policies, yet *how* they achieve this outcome is quite different. The threat of job losses essentially punishes regulatory action, whereas the offering of incremental concessions enables regulatory inaction by accommodating some of the public health demands in an industry initiative.

More directly, in business terms, the food industry’s strategies of appeasement and co-option (its “part of the solution” strategy) have paid market and political dividends. In the case of product reformulation and diversification, they have expanded the market share and revenues of powerful companies.^130^ Incremental changes to products or business practices (such as reducing the sugar in some products or restricting marketing to children in certain jurisdictions) help to diffuse challenges to the status quo and to reduce pressures for transformative change.^64,126,131,132^ The industry’s development and promotion of self-regulation as well as their participation in policy-making also acquire and secure private rule-making power and enable industry governance of the food system.^53,121^ Corporate accommodations and concessions can also improve the industry’s relationship with influential stakeholders in government and public health, as we can see with the food industry in South Africa.^69^ Lastly, the potential for the industry’s “part of the solution” strategy to persuade public health advocates to collaborate with the industry or view them as potential partners can fragment the public health community. The absence of a strong public health alliance has important consequences for political influence, as interest group cohesion is a predictor of political influence.^133-135^ The lack of a coordinated public health voice gives the food industry the opportunity to dominate issue framing in the media.^136^ This suggests that in addition to bolstering the political resources of the food industry, the strategies of appeasement and co-option can also diminish the political resources of the industry’s opposition by fragmenting it or making it seem redundant or overbearing.

Despite this, there is a clear consensus that the industry’s offensive and defensive strategies conflict with public health interests and still continue to this day despite the changes discussed above. In Gramscian terms, hegemony is never complete and is always subject to new challenges.^137^ The absence of corporate transparency about these strategies and the lengths companies go to hide these political strategies from public view demonstrate the industry’s awareness that these strategies reflect poorly on their public image. In contrast, the industry’s appeasement, co-option and partnership strategies have fostered greater ambivalence from public health and government stakeholders. Some government and public health stakeholders support industry efforts to be “more responsible” and endorse the development of public-private partnerships.^138-140^ Others have expressed scepticism over whether corporate actions would match corporate rhetoric. Indeed, the absence of promised change, and continued marketing of harmful products have produced a push-back in terms of the leniency and legitimacy offered to food corporations. What were often benign perspectives on their Road to Damascus transitions have shifted in some high-profile cases. For example, while the WHO outlined a range of “recommended actions” for the private sector in its 2004 *Global Strategy on Diet, Physical Activity and Health* and referred to the private sector as “partners” in its *2008-2013 Action Plan for Noncommunicable Diseases*, its 2016 report on *Fiscal policies for diet and the prevention of noncommunicable diseases* focused instead on strategies to counteract industry opposition to public health policies.^136,141,142^

## Conclusion

Corporate strategies of appeasement, co-option and partnership can be characterised by the giving of concessions, the emphasis on corporate responsibility and good behaviour, and projecting the zeitgeist of corporations being more engaged, open and involved in partnerships designed to promote healthy diets or regulate the more harmful effects of certain food products. The co-option of food governance levers and institutions and the perception of food corporation legitimacy in the Gramscian sense are also understood to be powerful tools of socio-political and cultural power. Food corporations have surely understood these as important strategically, and such strategic shifts would have been planned, learned through observation, or otherwise filtered through the global culture of corporate experience. Part of this logic is the transition of industry under capitalism, which mutated from the robber baron dynamics of the late nineteenth and early Twentieth centuries, to the Fordist models of the mid-Twentieth century, through to modern benevolent practices and forms of public engagement witnessed by CSR, philanthrocapitalism, corporate foundations, self-regulation and so on.^92^ All these new friendly faced variants of capitalism mask, perhaps, the growth of transnational oligopolies, financialization and deregulation, workforce precarity, austerity, all under public-private neoliberal global governance and truly transnational regulatory capture.^143^

 Ultimate, all this is about the relationship between market strategy – that concerned with preserving market position and profits – and non-market strategies – those being the political and regulatory environments that permit or inveigh on business. Food corporations have recognised that different non-market strategies were required in the face of an increasingly hostile regulatory environment, and shifted gear in their relationships with institutions, public health and the broader public. They have at the same time regeared product lines, continued to sell harmful products where they can, and made yet more profits. Politically and commercially it is a power play that appears to be succeeding unless it continues to be challenged in perhaps new and equally strategic ways.

## Ethical issues

 Not applicable.

## Competing interests

 All authors have denied any financial or other relationship that might lead to a conflict of interest.

## Authors’ contributions

 JLN and OW developed the concept for the paper and both collaborated on the first draft of the paper and subsequent iterations. All authors read and approved the final manuscript.

## Authors’ affiliations

^1^Centre for Health Policy, Melbourne School of Population and Global Health, University of Melbourne, Melbourne, Australia. ^2^School of Politics and International Studies, University of Leeds, Leeds, UK.
